# Supplementary material for: Integrated Analysis of the Lung Microbiome and Metabolome Reveals Associations Between Amino Acid Metabolism and Pulmonary Fibrosis in a Bleomycin-Induced Mouse Model
Source: Int J Mol Sci. 2026 Jun 30;27(13):5895. doi: 10.3390/ijms27135895 (PMC13362081; doi:10.3390/ijms27135895)
Supplement: Supplementary file 1 [file ijms-27-05895-s001.zip › result/5.MetKeggEnrichment/B_14d.vs.C_14d/B_14d.vs.C_14d_all_KEGG_map/KEGG.map.html]

Pathway Enrichment


  

# The most enriched pathway terms of B\_14d.vs.C\_14d\_all

Statistic method: hypergeometric test

FDR correction method: Benjamini and Hochberg

| MapID | MapTitle | Pvalue | AdjustedPv | x | y | n | N | EnrichDirect | GeneIDs |
| --- | --- | --- | --- | --- | --- | --- | --- | --- | --- |
| map00480 | Glutathione metabolism | 0.00777646393869367 | 0.225517454222116 | 3 | 8 | 15 | 255 | Over | Com\_3989\_pos Com\_50\_pos Com\_55\_neg |
| map00020 | Citrate cycle (TCA cycle) | 0.0292080771764229 | 0.282344746038755 | 2 | 5 | 15 | 255 | Over | Com\_13\_neg Com\_632\_neg |
| map00120 | Primary bile acid biosynthesis | 0.0292080771764229 | 0.282344746038755 | 2 | 5 | 15 | 255 | Over | Com\_1187\_pos Com\_506\_neg |
| map00053 | Ascorbate and aldarate metabolism | 0.0423184504106963 | 0.284313725490196 | 2 | 6 | 15 | 255 | Over | Com\_466\_pos Com\_55\_neg |
| map04713 | Circadian entrainment | 0.0588235294117647 | 0.284313725490196 | 1 | 1 | 15 | 255 | Over | Com\_187\_neg |
| map04918 | Thyroid hormone synthesis | 0.0588235294117647 | 0.284313725490196 | 1 | 1 | 15 | 255 | Over | Com\_3989\_pos |
| map00270 | Cysteine and methionine metabolism | 0.073713879368278 | 0.302525209299431 | 2 | 8 | 15 | 255 | Over | Com\_1120\_pos Com\_719\_pos |
| map00630 | Glyoxylate and dicarboxylate metabolism | 0.0915616008379399 | 0.302525209299431 | 2 | 9 | 15 | 255 | Over | Com\_13\_neg Com\_632\_neg |
| map01200 | Carbon metabolism | 0.110579218684424 | 0.302525209299431 | 2 | 10 | 15 | 255 | Over | Com\_122\_neg Com\_13\_neg |
| map00472 | D-Arginine and D-ornithine metabolism | 0.114404817044928 | 0.302525209299431 | 1 | 2 | 15 | 255 | Over | Com\_50\_pos |
| map01100 | Metabolic pathways | 0.121138541863729 | 0.302525209299431 | 11 | 129 | 15 | 255 | Over | Com\_1120\_pos Com\_122\_neg Com\_13\_neg Com\_187\_neg Com\_466\_pos Com\_506\_neg Com\_50\_pos Com\_55\_neg Com\_632\_neg Com\_65\_neg Com\_719\_pos |
| map01210 | 2-Oxocarboxylic acid metabolism | 0.151426412176217 | 0.302525209299431 | 2 | 12 | 15 | 255 | Over | Com\_50\_pos Com\_632\_neg |
| map00620 | Pyruvate metabolism | 0.166910460303134 | 0.302525209299431 | 1 | 3 | 15 | 255 | Over | Com\_13\_neg |
| map00660 | C5-Branched dibasic acid metabolism | 0.166910460303134 | 0.302525209299431 | 1 | 3 | 15 | 255 | Over | Com\_632\_neg |
| map00981 | Insect hormone biosynthesis | 0.166910460303134 | 0.302525209299431 | 1 | 3 | 15 | 255 | Over | Com\_527\_pos |
| map04979 | Cholesterol metabolism | 0.166910460303134 | 0.302525209299431 | 1 | 3 | 15 | 255 | Over | Com\_506\_neg |
| map00430 | Taurine and hypotaurine metabolism | 0.216499123380328 | 0.330446030422607 | 1 | 4 | 15 | 255 | Over | Com\_506\_neg |
| map00900 | Terpenoid backbone biosynthesis | 0.216499123380328 | 0.330446030422607 | 1 | 4 | 15 | 255 | Over | Com\_527\_pos |
| map04216 | Ferroptosis | 0.216499123380328 | 0.330446030422607 | 1 | 4 | 15 | 255 | Over | Com\_3989\_pos |
| map00030 | Pentose phosphate pathway | 0.307522571835426 | 0.445907729161368 | 1 | 6 | 15 | 255 | Over | Com\_122\_neg |
| map00140 | Steroid hormone biosynthesis | 0.349238079556184 | 0.468360578349597 | 1 | 7 | 15 | 255 | Over | Com\_2449\_pos |
| map01230 | Biosynthesis of amino acids | 0.355308024954867 | 0.468360578349597 | 2 | 21 | 15 | 255 | Over | Com\_1120\_pos Com\_50\_pos |
| map00220 | Arginine biosynthesis | 0.388598679583028 | 0.489972248169905 | 1 | 8 | 15 | 255 | Over | Com\_50\_pos |
| map00250 | Alanine, aspartate and glutamate metabolism | 0.425728314426164 | 0.514421713264948 | 1 | 9 | 15 | 255 | Over | Com\_65\_neg |
| map04080 | Neuroactive ligand-receptor interaction | 0.46074488061969 | 0.534464061518841 | 1 | 10 | 15 | 255 | Over | Com\_187\_neg |
| map00330 | Arginine and proline metabolism | 0.493760500173587 | 0.550732865578231 | 1 | 11 | 15 | 255 | Over | Com\_50\_pos |
| map00380 | Tryptophan metabolism | 0.52488178090062 | 0.554210066030212 | 1 | 12 | 15 | 255 | Over | Com\_187\_neg |
| map02010 | ABC transporters | 0.554210066030212 | 0.554210066030212 | 1 | 13 | 15 | 255 | Over | Com\_50\_pos |
| map04976 | Bile secretion | 0.554210066030212 | 0.554210066030212 | 1 | 13 | 15 | 255 | Over | Com\_506\_neg |
